# *Tylocinum* is no longer monotypic: *Tylocinumbrevisporum* sp. nov. (Boletales, Boletaceae) from northern Thailand

**DOI:** 10.3897/BDJ.9.e75907

**Published:** 2021-12-22

**Authors:** Bhavesh Raghoonundon, Naveed Davoodian, Monthien Phonemany, Olivier Raspé

**Affiliations:** 1 Center of Excellence in Fungal Research, Mae Fah Luang University, Chiang Rai, Thailand Center of Excellence in Fungal Research, Mae Fah Luang University Chiang Rai Thailand; 2 School of Science, Mae Fah Luang University, Chiang Rai, Thailand School of Science, Mae Fah Luang University Chiang Rai Thailand; 3 National Herbarium of Victoria, Royal Botanic Gardens Victoria, Melbourne, Victoria, Australia National Herbarium of Victoria, Royal Botanic Gardens Victoria Melbourne, Victoria Australia

**Keywords:** new species, Boletaceae, Leccinoideae, molecular phylogeny, taxonomy, Thailand

## Abstract

**Background:**

*Tylocinum* Y.C. Li & Zhu L. Yang 2016 is a Boletaceae genus belonging in subfamily Leccinoideae. It was described in 2016 from China and, prior to this study, it contained only one species, *T.griseolum* Y.C. Li & Zhu L. Yang 2016. During our survey of Boletaceae from Thailand, we collected some specimens that could be identified as a *Tylocinum* species, different from *T.griseolum*.

**New information:**

The bolete specimens, collected in forests dominated by Dipterocarpaceae and Fagaceae in northern Thailand, are described as *Tylocinumbrevisporum* Raghoonundon & Raspé sp. nov. Macroscopic and microscopic descriptions with illustrations are provided, as well as a 3-gene phylogeny, which confirms the new taxon’s position in *Tylocinum*. *Tylocinumbrevisporum* differs from the only other known *Tylocinum* species (*T.griseolum)* by its brownish-grey colour, greyish-orange to brownish-orange colour change in the hymenophore when bruised, smaller pores (≤ 0.5 mm), longer tubes (up to 6 mm long), shorter and narrower basidiospores, longer and broader basidia and longer pleurocystidia relative to cheilocystidia. *T.brevisporum* is the second species from the genus *Tylocinum* and the only one to be found outside China thus far.

## Introduction

*Tylocinum* Y.C. Li & Zhu L. Yang 2016, is a monotypic genus of ectomycorrhizal (ECM) boletes (Boletaceae, Boletales, Agaricomycetes, Basidiomycota, Fungi). Typical characters of the genus are its dark scabrous stipe surface, white to pallid unchanging context in the pileus and stipe, white to pallid hymenophore, trichodermium pileipellis and smooth basidiospores (Wu et al. 2016). The type species *Tylocinumgriseolum* Y.C. Li & Zhu L. Yang 2016, was originally described from China and was the only species known from this genus at the time. The phylogenetic analyses by Wu et al. (2016) showed that *Tylocinum* forms a separate clade from all other generic clades in the subfamily Leccinoideae.

The plant family Dipterocarpaceae includes many species of large trees that are often dominant in the tropical and subtropical lowlands of Southeast Asia, where the species diversity of Dipterocarpaceae is highest (Ashton 1982, Hamilton et al. 2019). Many Dipterocarpaceae are well known to be ECM, symbiotically associating with various ECM fungi, including mushroom-forming species (Watling et al. 2002, Yuwa-Amornpitak et al. 2006, Brearley 2012). Several new genera and species of boletes have recently been documented from tropical dipterocarp forest (Desjardin et al. 2009, Neves et al. 2012, Hosen et al. 2013, Halling et al. 2014, Raspé et al. 2016, Wu et al. 2016, Vadthanarat et al. 2019, Chuankid et al. 2019). Members of the Fagaceae, which also form ECM associations, co-occur with dipterocarps in Southeast Asia (Smith et al. 2008), which promotes higher mycodiversity and ECM colonisation in those tropical forest ecosystems (Corrales et al. 2018).

In this study, we describe a new species of *Tylocinum* from dry dipterocarp forests of northern Thailand, with description, illustrations and molecular phylogenetic analyses of a multi-gene DNA sequence dataset (*atp*6, *tef*1 and *rpb*2).

## Materials and methods

### Specimens collected

Fresh basidiomata were collected during the rainy season (2019) from Chiang Mai and Chiang Rai Provinces, orthern Thailand. The basidiomata were photographed on-site and wrapped in aluminium foil. The descriptions of the macroscopic features were made on the same day, after which the basidiomata were dried in an electric drier at 45–50°C. Specimens were deposited in the Mae Fah Luang University (MFLU) or CMUB Herbaria.

### Ecological, morphological and taxonomic study

The habitat, locality information and macro-chemical reactions on fresh basidiomata were recorded. Spore prints were taken for each collection. Colour codes were given using Kornerup and Wanscher (1978) as a guide. Microscopic characters were studied in the dried specimens. The following mounting solutions were used to observe the tissues: 10% aqueous potassium hydroxide (KOH) or 28–30% ammonium hydroxide (NH_4_OH) solutions or 1% ammoniacal Congo red solution. The microscopic structures were studied at magnifications of 60× and 100×, photographed with a calibrated Nikon Y-TV55 camera, fitted to a Nikon DIC microscope. A total of 60 basidiospores, 30 basidia, 30 pleurocystidia, 30 cheilocystidia and 30 terminal cells and 30 hyphae for both the pileipellis and stipitipellis were measured. The dimensions of the microscopic features are presented in the following format: (a–) b–c–d (−e), in which c represents the average, b the 5^th^ percentile, d the 95^th^ percentile and a and e the minimum and maximum values, respectively. Q, the length/width ratio for the spores, is presented in the same format. All microscopic features were drawn by free hand, using a drawing tube. Faces of Fungi (Jayasiri et al. 2015) and MycoBank numbers are provided for the new species.

### DNA extraction, PCR amplification and sequencing

Genomic DNA was extracted from CTAB-preserved tissues or dry specimens (ca. 10 mg) using a CTAB isolation procedure, adapted from Doyle and Doyle (1990). The *atp*6, *tef*1 and *rpb*2 gene regions were amplified by polymerase chain reaction (PCR). For amplification of *atp*6, the primers ATP6-1M40F and ATP6-2M were used (Raspé et al. 2016). EF1-983F and EF1-2218R (Rehner and Buckley 2005) were used to amplify *tef*1 and bRPB2-6F and bRPB2-7.1R (Matheny 2005) were used to amplify *rpb*2. The PCR amplification, purification and sequencing of *atp*6, *rpb*2 and *tef*1 were used following the procedure from Raspé et al. (2016).

### Sequence alignment and phylogenetic analysis

The sequences were assembled using Geneious 8 (Biomatters). The Basic Local Alignment Search Tool (BLAST) (https://blast.ncbi.nlm.nih.gov/Blast.cgi) from GenBank was used to find the closest matches to the sequences. Reference sequences (Table 1) were downloaded and aligned using MAFFT v. 7 (Katoh and Standley 2013; http://mafft.cbrc.jp/alignment/server/). Then, the concatenated three-gene matrix was prepared.

All analyses were done on the CIPRES Science Gateway (https://www.phylo.org; Miller et al. 2012). Maximum Likelihood (ML) phylogenetic tree inference was done using RAxML-HPC2 v.8.2.10 (Stamatakis 2006), using the GTRCAT model of sequence evolution with 25 categories. Three *Lanmaoa* species and three *Baorangia* species were selected as outgroup. Four partitions were defined: *atp*6, *tef*1 exons, *rpb*2 exons and introns. Statistical support of the clades was obtained using 1,000 rapid bootstrap replicates.

Using jModeltest2 (Darriba et al. 2012) on XSEDE via the CIPRES Science Gateway, the best-fit model of substitution for analysis in MrBayes was estimated for each gene, based on the Bayesian Information Criterion (BIC). GTR + I + G for *atp*6 and introns, SYM + I + G for *tef*1 exons and K80 + I + G for *rpb*2 exons were selected as the best fit models. Partitioned Bayesian analysis was performed with MrBayes 3.2.7a (Ronquist et al. 2012). Two runs of four cold and one heated chains were run for 1,000,000 generations and sampled every 200 generations. The average standard deviation of split frequencies was 0.005106 at the end of the runs. The burn-in phase (25%) was estimated by checking the stationarity in the plot generated by the sump command.

## Taxon treatments

### Tylocinum brevisporum

Raghoonundon & Raspé sp. nov.

64D99B61-7573-598D-8BE9-02D0AC73258B

MB841102

FoF 10255

#### Materials

- **Type status:** Holotype. **Taxon:** kingdom: Fungi; phylum: Basidiomycota; class: Agaricomycetes; order: Boletales; family: Boletaceae; genus: Tylocinum; specificEpithet: *brevisporum*; taxonRank: species; **Location:** country: Thailand; stateProvince: Chiang Rai Province, Chang Wat, Doi Pui; verbatimElevation: 730 m; verbatimCoordinates: 19°48'50"N, 99°51'57"E; **Identification:** identifiedBy: Bhavesh Raghoonundon; **Event:** eventDate: 20 August 2019; **Record Level:** institutionID: MFLU 21-0144; institutionCode: Mae Fah Luang University Herbarium; collectionCode: BR137
- **Type status:** Other material. **Taxon:** kingdom: Fungi; phylum: Basidiomycota; class: Agaricomycetes; order: Boletales; family: Boletaceae; genus: Tylocinum; specificEpithet: *brevisporum*; taxonRank: species; **Location:** country: Thailand; stateProvince: Chiang Mai Province, Mueang District; verbatimElevation: 450 m; verbatimCoordinates: 18°48'40"N, 98°56'31"E; **Identification:** identifiedBy: Olivier Raspé; **Event:** eventDate: 18 May 2015; **Record Level:** institutionID: CMU-B OR622; collectionID: OR622; institutionCode: Chiang Mai University Herbaria

#### Description

**Figure 1.**
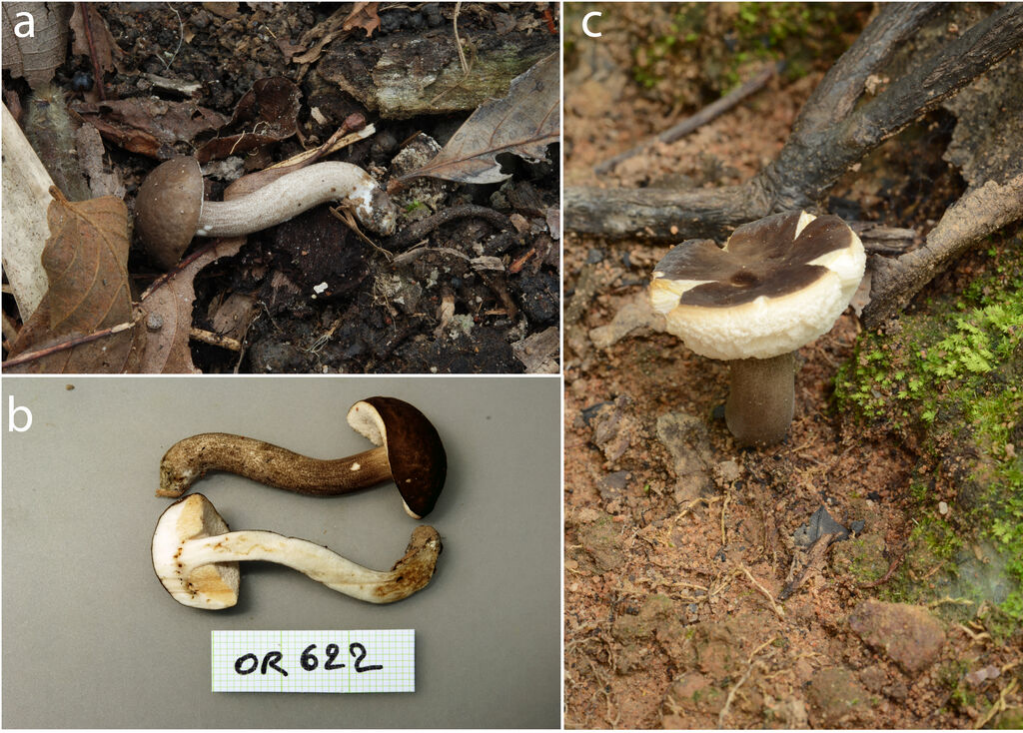
Photograph of *Tylocinumbrevisporum* sp. nov. **a, b** Basidioma of specimen OR622; **c** Basidioma of the holotype (BR 137).

**Basidiomata** pileo-stipitate, small to medium-sized (Fig. 1). **Pileus** (1.5–)2.0–2.5 cm in diameter, convex when young, becoming plano-depressed with age; margin deflexed to uplifted, surface finely tomentose, dull and dry, at first brown (7E4) to greyish-brown (8E3–8F4), becoming paler (8D3) near the margin with age; **context** 3–5 mm thick halfway to the margin, soft and fleshy, off-white, slightly browning on exposure. **Stipe** central, cylindrical, (3.4–)4.9–6.5 cm × 0.6–1.3 cm, surface even, dull and dry, scabrous, covered with granular squamules (dotted-verrucose), brownish-grey (7E2–8E2) when young to reddish-brown (8E5) to dark brown (8F5) with age, no colour change when bruised, basal mycelium off-white; **context** solid, fleshy, off-white, reddish-brown to dark brown near the stipe base (8F7) and in worm wounds, slightly browning on exposure. **Hymenophore** tubulate, subventricose, adnexed, slightly depressed around apex of the stipe, greyish-orange to brownish-orange when bruised. **Tubes** 3–6 mm long halfway to the margin, off-white, easily separable from one another. **Pores** ≤ 0.5 mm wide at mid-radius, regularly arranged, angular, off-white, turning brown to dark brown (8E5–8F5) when bruised. **Odour** fungoid. **Taste** bitter. **Spore print** not obtained.

**Figure 2.**
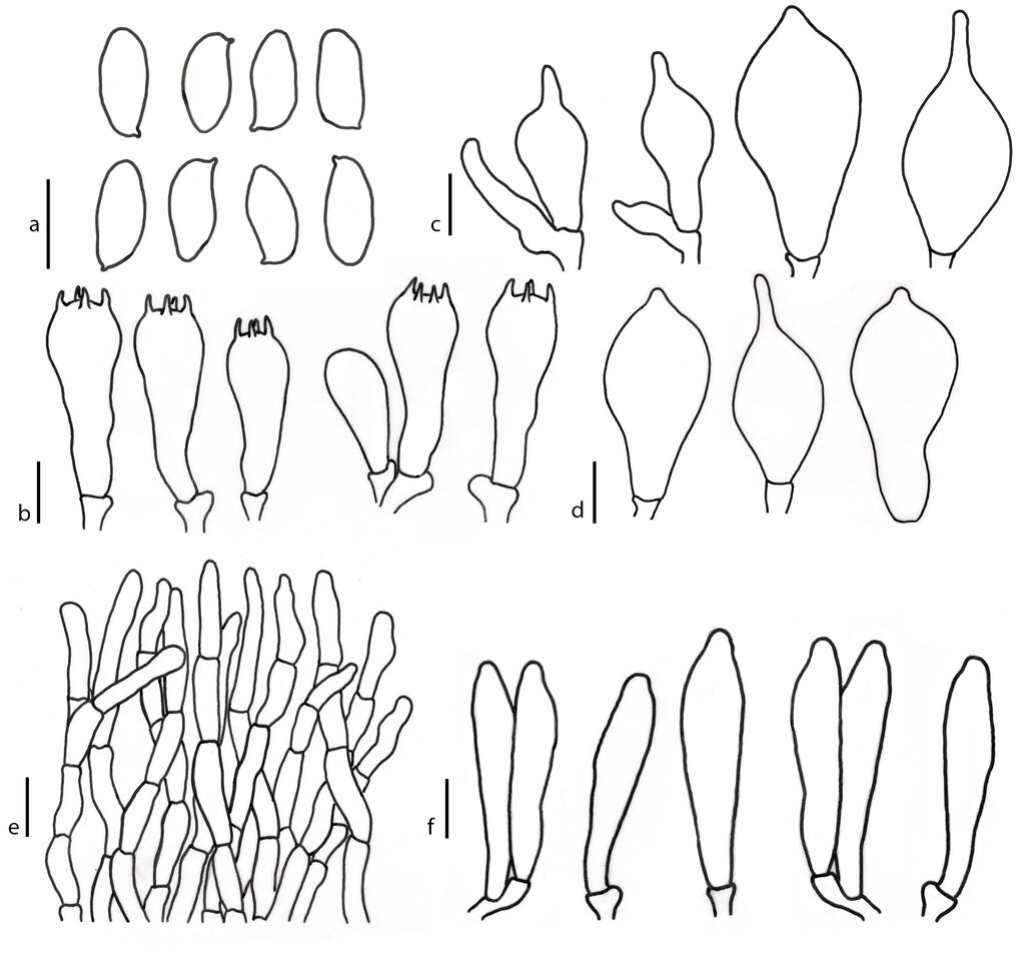
Microscopic features of *Tylocinumbrevisporum*; **a** Basidiospores; **b** Basidia; **c, d** Caulocystidia; **e** Pleurocystidia; **f** Cheilocystidia; **g** Pileipellis. Scale bars: a, b, c, d, f = 10 µm, e = 20 µm, g = 50 µm.

**Basidiospores** (6.7–)7.5–10–11.7(–11.8) × (3.1–)3.5–4.7–5.8(–5.9) µm (n = 50) *Q* = (1.7–) 1.79–2.15–2.5 (–2.61), ellipsoid in central view, oblong to subcylindrical in side view, smooth under light microscope, yellowish to brownish in KOH (Fig. 2). **Basidia** 4-spored, (27–)27–37.4–54(–54) × (9–)9–12.3–19(–19) µm, clavate, yellowish to brownish in KOH, sterigmata up to 3 µm long. **Cheilocystidia** (19–)19.3–25.5–33(–35) × (4–)4.1–6–8.2(–8.5) µm, frequent, fusiform, thin-walled, yellowish to brownish hyaline in KOH and NH_4_OH. **Pleurocystidia** (40­–)41–53–69(–70) × (8–)7.4–12–16.6(–17) µm, thin-walled, fusiform to broadly fusiform with a long pedicel and sharp apex, occasionally containing yellowish inclusions, yellowish to brownish hyaline in KOH and NH_4_OH. **Hymenophoral trama** boletoid, elements smooth, cylindrical, hyaline, 5–10 µm wide. **Pileipellis** a trichodermium, hyphae terminations with 3–4 cells that are 5–11 µm wide and terminal cells 31–48 µm × 6–10 µm, colourless to slightly brownish in KOH. **Pileus trama** composed of interwoven hyaline hyphae 5–9 µm wide. **Stipitipellis** a disrupted hymeniderm with hyphae 3.7–7.4 µm wide, colourless to slightly brownish in KOH and caulocystidia (24–)24.5–35–47(–48) × (9–)9.2–12.4–16.9(–17) µm, thin-walled, clavate to broadly clavate with a sharp apex, yellowish to brownish hyaline in KOH and NH_4_OH. **Stipe trama** composed of cylindrical, hyaline, interwoven hyphae 3.7–7.4 µm wide. **Clamp connections** absent.

#### Diagnosis

This species is distinguished from *Tylocinumgriseolum* by its greyish-brown colour, greyish-orange to brownish-orange colour change in the hymenophore when bruised, smaller pores (≤ 0.5 mm) and longer tubes (up to 6 mm long). Additionally, the basidiospores are shorter and narrower compared to *T.griseolum* and the basidia are slightly longer and broader. Furthermore, the pleurocystidia of *Tylocinumbrevisporum* are longer than its cheilocystidia.

#### Etymology

Epithet “*brevisporum*”; from the Latin words *brevi* (short) and *sporae* (spores), referring to the shorter spores of this species compared to *Tylocinumgriseolum*.

#### Distribution

Thus far known only from northern Thailand.

#### Ecology

Solitary, in tropical forest dominated by Dipterocarpaceae (*Dipterocarpus* spp. and *Shorea* spp.), with some Fagaceae (*Quercus* spp., *Lithocarpus* spp. and *Castanopsiscalathiformis*).

#### Notes

Morphologically, *Tylocinumbrevisporum* is similar to *Tylocinumgriseolum*, with which it shares the overall grey colour of the basidiomata and dark scabrous stipe surface. However, *Tylocinumbrevisporum* is more brownish as compared to the grey *Tylocinumgriseolum*. In addition, Wu et al. (2016) mentioned no discolouration in the context of *Tylocinumgriseolum*. The context of *Tylocinumbrevisporum* becomes slightly brown when bruised. The hymenophore of *T.brevisporum* changes to greyish-orange to brownish-orange when bruised as compared to the unchanging hymenophore of *T.griseolum*. Moreover, *T.griseolum* has relatively larger pores (up to 1.5 mm) than that of *T.brevisporum* (< 0.5 mm). The tubes in *T.griseolum* are also shorter than those of *T.brevisporum*.

The basidiospores of *Tylocinumbrevisporum* [(6.7–)7.5–10–11.7(–11.8) × (3.1–)3.5–4.7–5.8(–5.9) µm, *Q* = (1.7–)1.79–2.15–2.5(–2.61)] are shorter and narrower than those of *Tylocinumgriseolum* [(11)12.0–14.5(16) × 4.5–5.5 µm Q = 2.60–3.22] from China. The basidia of *T.brevisporum* [(27–)27–37.4–54(–54) × (9–)9–12.3–19(–19) µm] are also slightly longer and broader than *T.griseolum* [30–45 × 10–12 µm]. Wu et al. (2016) reported that, for *T.griseolum*, the pleurocystidia and cheilocystidia are similarly-sized. In *T.brevisporum*, the pleurocystidia are longer than the cheilocystidia. Phylogenetically, *T.brevisporum* clusters with *T.griseolum*, together forming a well-supported clade (MLB/BPP = 93/1.00) i.e. the genus *Tylocinum*.

## Analysis

### Phylogenetic analysis

**Figure 3.**
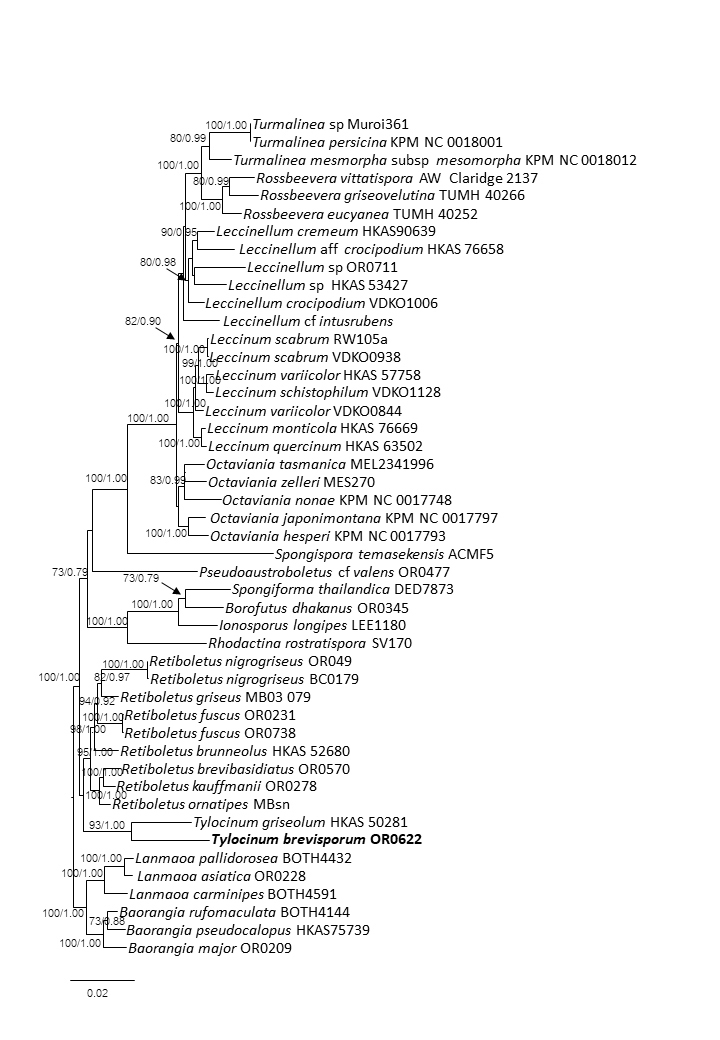
Maximum Likelihood phylogenetic tree inferred from the three-gene dataset (atp6, rpb2, tef1). The three *Lanmaoa* and three *Baorangia* species were used as outgroup taxa. Maximum Likelihood Bootstrap (MLB, left) ≥ 70% and Bayesian Posterior Probabilities (BPP, right) ≥ 0.95 are shown above supported branches. The new species is in bold.

The concatenated gene dataset comprised 47 terminals. The final alignment contained 121 sequences (38 for *atp*6, 46 for *tef*1, 37 for *rpb*2) and was 2,676 characters long, including gaps. Both ML and Bayesian analyses produced the same tree topology; thus, only the ML tree is shown with both Maximum Likelihood Bootstrap (MLB) and Bayesian Posterior Probabilities (BPP) values. In the analyses, the new species *Tylocinumbrevisporum* shared a sister relationship with the type species *Tylocinumgriseolum* (Fig. 3), providing strong statistical support (MLB = 93 and BPP = 1.00) for the genus *Tylocinum* (Leccinoideae). The *atp*6 sequence of the holotype (BR 137) was 100% identical to OR622.

## Discussion

Boletales is a globally-distributed order of fungi, comprising morphologically diverse groups (Binder and Hibbett 2006, Wu et al. 2016), with ECM, ligninolytic, saprobic and mycoparasitic members (Binder and Hibbett 2006, Kirk et al. 2008). Thorough morphological and phylogenetic analyses of the order has led to the discovery of new genera and other taxa (e.g. Binder and Bresinsky 2002, Wu et al. 2014, Zhu et al. 2015, Wu et al. 2016, Orihara et al. 2016, Vadthanarat et al. 2019, Zhang et al. 2019). Boletaceae Chevall. 1826 is a morphologically diverse family currently comprising of 94 genera distributed amongst seven subfamilies (Binder and Hibbett 2006, Wu et al. 2014, Wu et al. 2016). The subfamily Leccinoideae was revealed by the phylogenetic analyses of Wu et al. (2014). Currently, this subfamily comprises fifteen genera, viz. *Binderoboletus* T.W. Henkel & M.E. Sm. 2016, *Borofutus* Hosen & Z.L. Yang 2012, *Chamonixia* Rolland 1899, *Ionosporus* Khmeln. 2018, *Kaziboletus* Iqbal Hosen & Zhu L. Yang 2021, *Leccinum* Gray 1821, *Lecinellum* Bresinsky & Manfr. Binder 2003, *Pseudoaustroboletus* Y.C. Li & Zhu L. Yang 2014, *Octavania*, *Retiboletus* Manfr. Binder & Bresinsky 2002, *Rossbeevera* T. Lebel & Orihara 2012, *Rhodactina* Pegler & T.W.K. Young 1989, *Spongiforma* Desjardin, Manfr. Binder, Roekring & Flegel 2009, *Spongispora* G. Wu, S.M.L. Lee, E. Horak & Z.L. Yang 2018, *Turmalinea* Orihara & N. Maek. 2015 and *Tylocinum*. Only ten of these genera are stipitate-pileate.

Our survey on the diversity of boletes in northern Thailand led to the discovery of a second species of *Tylocinum* (the focus of the present study), being found in tropical forests dominated by Dipterocarpaceae, which have been reported as ECM hosts for Boletaceae (Desjardin et al. 2009, Halling et al. 2014, Wu et al. 2018, Vadthanarat et al. 2019). According to Wu et al. (2016), the white to dirty white hymenophore of *Tylocinum* is similar to that of *Tylopilus* Karst. 1881 when young, while the verrucose stipe surface is similar to *Leccinum*. The stipe surface of *Tylocinum* is dotted-verrucose, which may give a more or less rough touch, but it does not produce markedly projecting scabers like in *Leccinum*. *Tylocinum* is also similar to *Tylopilus*, but there are some morphological differences between the two genera. *Tylopilus* species usually produce larger basidiomata and have minutely and densely tomentose to dotted-tomentose, but never dotted-verrucose, stipitipellis. Moreover, some *Tylopilus* species have reticulate stipe, whereas, in *Tylocinum*, the stipe is at most longitudinally venose near the apex. As the diversity of Boletaceae in Thailand is high and remains understudied (e.g. Vadthanarat et al. 2021), further studies may uncover additional species of *Tylocinum* or related taxa.

## Supplementary Material

XML Treatment for Tylocinum brevisporum

## Figures and Tables

**Table 1.** List of collections used for DNA analyses, with origin, GenBank accession numbers and reference(s).

**Species**	**Voucher**	**Origin**	***atp* 6**	***tef* 1**	***rpb* 2**	**References**
*Baorangiamajor*	OR0209	Thailand	MG897421	MG897431	MG897441	Phookamsak et al. (2019)
*Baorangiapseudocalopus*	HKAS75739	China	–	KJ184570	KM605179	Wu et al. (2015)
*Baorangiarufomaculata*	BOTH4144	USA	MG897415	MG897425	MG897435	Phookamsak et al. (2019)
*Borofutusdhakanus*	OR0345	Thailand	MH614660	MH614709	MH614755	Vadthanarat et al. (2018)
*Ionosporuslongipes*	LEE1180	Malaysia	MT085461	MT085471	MH712031	Khmelnitsky et al. (2019)
*Lanmaoaasiatica*	OR0228	China	MH614682	MH614730	MH614777	Vadthanarat et al. (2019)
*Lanmaoacarminipes*	BOTH4591	USA	MG897419	MG897429	MG897439	Phookamsak et al. (2019)
*Lanmaoapallidorosea*	BOTH4432	USA	MG897417	MG897427	MG897437	Phookamsak et al. (2019)
*Leccinummonticola*	HKAS76669	China	–	KF112249	KF112723	Wu et al. (2014)
*Leccinumquercinum*	HKAS63502	China	–	KF112250	KF112724	Wu et al. (2014)
*Leccinumscabrum*	RW105a	Belgium	KT823979	KT824045	KT824012	Raspé et al. (2016)
*Leccinumscabrum*	VDKO0938	Belgium	MG212549	MG212593	MG212635	Vadthanarat et al. (2018)
*Leccinumschistophilum*	VDKO1128	Belgium	KT823989	KT824055	KT824022	Raspé et al. (2016)
*Leccinumvariicolor*	HKAS57758	China	–	KF112251	KF112725	Wu et al. (2014)
*Leccinumvariicolor*	VDKO0844	Belgium	MG212550	MG212594	MG212636	Vadthanarat et al. (2018)
Leccinellumaff.crocipodium	HKAS76658	China	–	KF112252	KF112728	Wu et al. (2014)
Lecinellumcf.intusrubens	OR0082	Thailand	MZ803019	MZ803024	MZ824749	This study
*Leccinellumcrocipodium*	VDKO1006	Belgium	KT823988	KT824054	KT824021	Raspé et al. (2016)
*Leccinellumcremeum*	HKAS90639	China	–	KT990781	KT990420	Wu et al. (2016)
*Leccinellum* sp.	HKAS53427	China	–	KF112253	KF112727	Wu et al. (2014)
*Leccinellum* sp.	OR0711	Thailand	MH614685	MH614733	MH614780	Vadthanarat et al. (2019)
*Octavianiahesperi*	KPM-NC 17793	Japan	KC552150	JN378422	–	Orihara et al. (2016)
*Octavianiajaponimontana*	KPM-NC 17797	Japan	KC552151	JN378425	–	Orihara et al. (2016)
*Octavianianonae*	KPM-NC 17748	Japan	KC552143	JN378403	–	Orihara et al. (2016)
*Octavianiatasmanica*	MEL 2341996	Australia	KC552156	JN378436	–	Orihara et al. (2012), Orihara et al. (2016)
*Octavianiazelleri*	MES270	USA	KC552161	JN378440	–	Orihara et al. (2012), Orihara et al. (2016)
Pseudoaustroboletuscf.valens	OR0477	China	MZ803020	MZ803025	MZ824750	This study
*Retiboletusbrevibasidiatus*	OR0570	Thailand	MT085469	MT085476	MT085479	Chuankid et al. (2021)
*Retiboletusbrunneolus*	HKAS 52680	China	–	KF112179	KF112690	Wu et al. (2014)
*Retiboletusfuscus*	OR0231	China	MG212556	MG212600	MG212642	Vadthanarat et al. (2018)
*Retiboletusfuscus*	OR0738	Thailand	MT085462	MT085472	MT085477	Chuankid et al. (2021)
*Retiboletusgriseus*	MB03-079	USA	KT823964	KT824030	KT823997	Raspé et al. (2016)
*Retiboletuskauffmanii*	OR0278	China	MG212557	MG212601	MG212643	Vadthanarat et al. (2018)
*Retiboletusnigrogriseus*	BC0179	Thailand	MT085464	MT085474	MT085478	Chuankid et al. (2021)
*Retiboletusnigrogriseus*	OR049	Thailand	KT823967	KT824000	KT824033	Raspé et al. (2016)
*Retiboletusornatipes*	MBsn	USA	MT219514	MT219516	MT219515	Chuankid et al. (2021)
*Rhodactinarostratispora*	SV170	Thailand	MG212560	MG212605	MG212645	Vadthanarat et al. (2018)
*Rossbeeveraeucyanea*	TUMH-40252	Japan	KC552116	KC552069	–	Orihara et al. (2016)
*Rossbeeveragriseovelutina*	TUMH-40266	Japan	KC552121	KC552073	–	Orihara et al. (2016)
*Rossbeeveravittatispora*	A.W. Claridge 2137	Australia	KC552105	KC552063	–	Orihara et al. (2016)
*Spongiformathailandica*	DED7873	Thailand	MG212563	KF030436	MG212648	Nuhn et al. (2013), Vadthanarat et al. (2018)
*Spongisporatemasekensis*	ACMF5	Singapore	MZ803018	MZ803023	MZ824748	This study
Turmalineamesomorphasubsp.mesomorpha	KPM-NC 18012	Japan	KC552139	KC552090	–	Orihara et al. (2016)
*Turmalineapersicina*	KPM-NC 18001	Japan	KC552130	KC552082	–	Orihara et al. (2016)
*Turmalinea* sp.	Muroi361	USA	DQ218885	DQ219224	DQ219046	Orihara et al. (2016)
*Tylocinumgriseolum*	HKAS50281	China	–	KF112284	KF112730	Wu et al. (2014)
*Tylocinumbrevisporum*	OR622	Thailand	MZ803021	–	MZ824751	This study
